# Safety and efficacy profile of Trastuzumab deruxtecan in solid cancer: pooled reanalysis based on clinical trials

**DOI:** 10.1186/s12885-022-10015-6

**Published:** 2022-08-26

**Authors:** Hanyue Xu, Hao Zhang, Wen Guo, Xi Zhong, Jing Sun, Tao Zhang, Zhoufeng Wang, Xuelei Ma

**Affiliations:** 1 Department of Biotherapy, West China Hospital and State Key Laboratory of Biotherapy, Sichuan University, Chengdu, 610041 Sichuan P.R. China; 2 Department of Ophthalmology, West China Hospital, Sichuan University, Chengdu, 610041 Sichuan P.R. China; 3 Department of Pancreatic Surgery, West China Hospital, Sichuan University, Chengdu, 610041 Sichuan P.R. China; 4 The Center of Gerontology and Geriatrics, West China Hospital, Sichuan University, Chengdu, 610041 PR China; 5 Department of Intensive Care Unit, West China HospitalSichuan University, Chengdu, 610041 PR China; 6 Integrated Traditional and Western Medicine Department, Qingdao Central Hospital, Qingdao University, Qingdao, Shandong 266042 P.R. China; 7 Institute of Respiratory Health, Frontiers Science Center for Disease-Related Molecular Networks, West China Hospital, Sichuan University, Chengdu, 610041 Sichuan P.R. China

**Keywords:** Trastuzumab deruxtecan (DS-8201a), Adverse events, Progression free survival, Human epidermal growth factor receptor 2, Breast cancer, Gastric cancer

## Abstract

**Purpose:**

This study aimed to explore the efficiency and safety of the new generation antibody-drug conjugate Trastuzumab deruxtecan (DS-8201a) in treating HER2-positive solid cancers.

**Method:**

By searching PubMed, Medline and Ovid for all clinical trials related to the safety and efficacy of DS-8201a. Event rates were calculated for all adverse events (AEs) to evaluate the safety of DS-8201a. Objective response rate (ORR) and progression-free survival (PFS) were summarized to assess the potency of DS-8201a.

**Result:**

The AEs with event rates greater than 30% regardless of grades were nausea, decreased appetite, vomiting, fatigue, anemia, decreased neutrophil count, alopecia and diarrhea. In the grade 3 or more, decreased neutrophil count, anemia and decreased white blood cell count were the only three AEs with event rates greater than 10% (20.3, 15.0 and 10.3%). The median PFS of patients with breast cancer, gastric cancer and other HER2-positive solid cancers were 9.0-22.1, 3.0-8.3 and 4.1-11.9 months. The median ORR was 37-79.9% in patients with breast and gastric cancer and 28.3-55% in patients with other HER2-positive cancers.

**Conclusion:**

DS-8201a plays an active role in treating HER2-positive cancers, especially breast and gastric cancer, which have HER2 amplification. The most common AEs of DS-8201a were related to gastrointestinal and hematological system. Decreased white blood cell count and appetite were the AEs occurred with high grades.

**Supplementary Information:**

The online version contains supplementary material available at 10.1186/s12885-022-10015-6.

## Introduction

Human epidermal growth factor receptor 2 (HER2) is one of the epidermal growth factor transmembrane receptor family. The amplification, mutation and overexpression of HER2 can promote the proliferation, adhesion, migration, differentiation and apoptosis of tumor cells and is associated with aggressive diseases [1]. Targeting HER2 is a burgeoning method for treating several kinds of HER2-positive tumors, including breast cancer, gastric cancer, and non-small cell lung cancer [2–4]. About 15-20% of breast cancer, 6 to 30% of advanced gastric or gastro-esophageal junction cancers, and 7 to 9% NSCLCs are HER2-positive [5–8]. Combination of anti-HER2 humanized monoclonal antibody and chemotherapy is the first line therapy recommended to patients with metastatic HER2-positive breast cancer, and the antibody-drug conjugate (ADC) trastuzumab emtansine is the standard second-line therapy [9, 10]. According to the phase 3 ToGA trial, trastuzumab is the first approved drug for anti-HER2 therapy in HER2-overexpressing gastric cancer [3]. However, breast cancer is still the disease that responds best to these drugs, which may account for the higher expression of HER2 in breast cancer [6].

ADC commonly has three components, an antibody, a linker and a payload cytotoxic agent [11]. The antibody is used to against the target antigen, the cytotoxic agents have standby effect, and the linker connects these two components [12]. Trastuzumab deruxtecan (DS-8201a) is a kind of ADCs and composed of a humanized anti-HER2 antibody, a potent topoisomerase I inhibitor (an exatecan derivative, DXd) and a tetrapeptide linker, which is stable in plasma and can be cleaved by cathepsin in tumor cells [13]. The anti-HER2 antibody in DS-8201a is a human monoclonal IgG1 and its amino acid sequence is the same as trastuzumab [13]. The drug-to-antibody ratio of DS-8201a is seven to eight, which is higher than that of trastuzumab emtansine (about four) [14]. Previous studies used trastuzumab, pertuzumab or trastuzumab emtansine to treat HER2-positive cancers, while some of them did not prolong overall survival of patients and some achieved high objective response rate (ORR) with severe drug resistance problem [3, 9, 15, 16].

As both the basic information and clinical results indicate DS-8201a as a potent effective drug for HER2-positive cancers, we found it necessary to summarized existing results. Hence, to explore the potency of DS-8201a in treating solid cancers, this study reviewed and pooled the results of all completed clinical studies.

## Method

### Search strategy

A comprehensive article review was made from 2016 to July 2022, as DS-8201a was first reported in 2016, by searching PubMed, Medline and Ovid for all clinical trials related to the safety and efficacy of DS-8201a and referring to the Cochrane guidelines of meta-analysis. The keywords used were “DS-8201a” or “Trastuzumab deruxtecan”. To guarantee no missing literature exists, the references of included studies were also screened. The research question of this study was investigating the potency of DS-8201a in treating patients with solid tumor, according to the adverse events (AEs) and survival condition data, like ORR, overall survival (OS), and progression free survival (PFS).

### Inclusion and exclusion criteria

Inclusion criteria were as follows: 1) clinical trials in any phase using DS-8201a as main strategy; 2) patients were with HER2-positive solid tumors; 3) the reported results included sufficient information of AEs and survival condition. Exclusion criteria was that studies: 1) in forms of review articles, laboratory articles, meta-analysis, or letters; 2) using other curing strategies without using DS-8201a alone; 3) without sufficient information about the survival or AEs of patients; 4) not in English version. Two authors selected articles independently and a third author with more experience was responsible for resolving divergences.

### Data extraction

Extracted data included: 1) basic information of studies: name of the first author, publication year, ClinicalTrials.gov number, study phase, sample size, tumor histological types, and treating regimes; 2) the characteristics of major AEs (mentioned in at least two trails), including AEs type, grades according to the National Cancer Institute Common Terminology Criteria for Adverse Events, number of patients with different AEs and survival parameters, like PFS, OS, ORR, time to response (TTR) and duration of response (DOR) of the patients.

### Statistical analysis

Comprehensive Meta-Analysis program 2 (Biostat, Englewood, NJ, USA) were used for meta-analysis. The proportion and derived 95% confidence interval (CI) were calculated for major AEs, and subgroups were divided based on the grades of AEs. The results were considered significant when the *p* value was less than 0.05. Random-effects model was used when *I*^2^ was larger than 50%.

### Study quality assessment

The quality of articles that made randomized controlled trials was assessed by Cochrane’s risk of bias tool (Review Manager 5.3), and for articles with non-randomized trials, methodological index for non-randomized studies (Slim et al., 2003) was used (Supplementary Table 1). All studies involved were evaluated by one author independently and inspected by another author.

## Results

### Study selection and characteristics

**Fig. 1:**
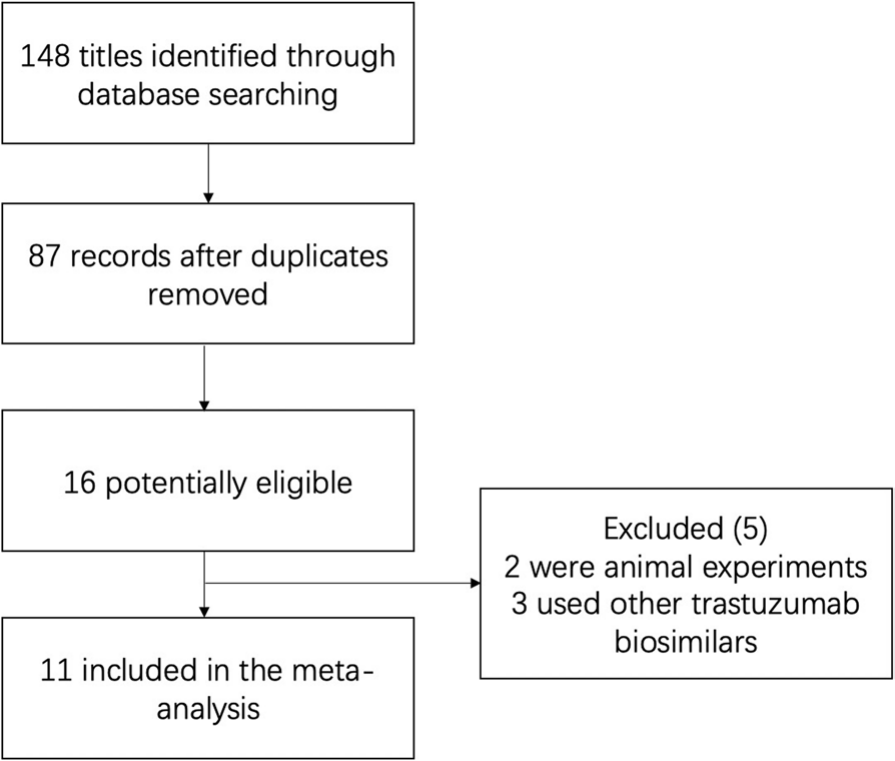
Flow chart of the article selection progress

After a systemic search in PubMed, Medline and Ovid, a total of 148 articles were obtained. Sixty-one articles were removed for duplication. By reading the titles and abstracts, 71 articles were excluded from 87 articles. Then, the full texts of all remained articles were read, 16 potential articles were reserved. Among them, two studies were animal experiments without clinical research [17, 18] and three of them used other trastuzumab biosimilars instead of Trastuzumab deruxtecan to treat patients [19–21]. Finally, 11 articles were defined as eligible and included in this meta-analysis [22–32]. This selection process was presented as a flow chart in Fig. 1. Among all included articles, five were phase I clinical trials, and 2 were phase II clinical trials. Six articles were single arm trials and only one trail compared the potency of DS-8201a with chemotherapy. The basic information of each included articles was listed in Table 1. A total of 587 patients were enrolled, of whom 528 were breast or gastric cancer and the remaining 59 were other solid cancers. According to the scores of methodological indexes, all included studies had high quality (Supplementary Table 1).

**Table 1:** Basic information of involved studies

Author	Year	ClinicalTrials.gov number	Sample size	Histology	Treatment regime	Study phase	Dose (mg/kg)
Doi	2017	NCT02564900	12	breast or gastric or gastro-oesophageal carcinomas	trastuzumab deruxtecan	phase 1	5·4 or 6·4
Park	2020	NCT02564900	184	HER2-positive meta- static breast cancer	trastuzumab deruxtecan	phase 2	5.4
Shitara	2019	NCT02564900	44	HER2-positive gastric or gastro-oesophageal junction cancer	trastuzumab deruxtecan	phase 1	5·4 or 6·4
Tamura	2019	NCT02564900	115	HER2-positive breast cancer	trastuzumab deruxtecan	phase 1	5·4 or 6·4
Modi	2020	NCT02564900	54	advanced/metastatic HER2-low–expressing breast cancer	trastuzumab deruxtecan	phase 1	5·4 or 6·4
Shitara	2020	NCT03329690	175 (119) ^a^	HER2-positive advanced gastric cancer	trastuzumab deruxtecan as compared with chemotherapy	phase 2	6·4
Tsurutani	2020	NCT02564900	59	HER2-expressing non-breast/non-gastric or HER2-mutant solid tumors	trastuzumab deruxtecan	phase 1	6·4
Siena	2021	NCT03384940	78	HER2-expressing metastatic colorectal cancer	trastuzumab deruxtecan	phase 2	4.4 or 5·4 or 6·4
Li	2022	NCT03505710	91	HER2-mutant non-small-cell lung cancer	trastuzumab deruxtecan	phase 2	6·4
Modi	2022	NCT03734029	557 (373) ^a^	HER2-low metastatic breast cancer	trastuzumab deruxtecan as compared with chemotherapy	phase 3	5·4
Cortés	2022	NCT03529110	524 (257) ^a^	HER2-positive metastatic breast cancer	trastuzumab deruxtecan as compared with trastuzumab emtansine	phase 3	5·4

^a^ The numbers in brackets represent the number of people in the trastuzumab deruxtecan group

### Safety

All included articles reported AEs and the grades of AEs were evaluated according to the National Cancer Institute’s Common Terminology Criteria for Adverse Events. The incidence of AEs in grades 3 or more and all grades were presented in Tables 2 and 3. The event rates were calculated as the rate of AE patients in all patients treated. AEs with event rates greater than 30% regardless of grades were nausea, decreased appetite, vomiting, fatigue, anemia, decreased neutrophil count, alopecia, and diarrhea. The event rates of nausea and decreased appetite were higher than 40, 72.3% (95% CI, 69.8 to 74.6%) and 44.3% (95% CI, 35 to 54.1%), respectively. In grades 3 or more, only decreased neutrophil count, anemia and decreased white blood cell count with relatively high rate, 20.3, 15.0 and 10.3% respectively. The detail of data analysis can be found in Supplementary Table 2.

**Table 2:** The adverse event rates (grades 3 or more) and 95% confidence interval of fixed model and random model in single-arm trials

Model	Group by	All grade				
		Event rate (%)	Lower limit (%)	Upper limit (%)	Z-Value	*p*-Value
Fixed	Abdominal distension	1.3	0.2	8.6	−4.303	0.000
Fixed	Abdominal pain	1.1	0.4	2.8	−9.443	0.000
Fixed	Alanine aminotransferase increased	1.7	0.9	3.4	−11.293	0.000
Fixed	Alopecia	0.6	0.3	1.3	−13.009	0.000
Fixed	Cholangitis	5.5	1.8	15.9	−4.765	0.000
Fixed	Constipation	0.7	0.3	1.5	−13.003	0.000
Fixed	Cough	1.0	0.3	2.7	−8.603	0.000
Fixed	Decreased lymphocyte count	8.6	6.1	11.8	−12.967	0.000
Fixed	Diarrhoea	2.1	1.4	3.1	−18.197	0.000
Fixed	Dizziness	0.9	0.1	5.9	−4.715	0.000
Fixed	Dysgeusia	0.7	0.1	3.6	−5.983	0.000
Fixed	Dyspnea	1.9	0.3	12.0	−3.933	0.000
Fixed	Ejection fraction decreased	0.7	0.1	3.6	−5.983	0.000
Fixed	Electrocardiogram QT prolonged	1.2	0.3	4.5	−6.236	0.000
Fixed	Epistaxis	0.6	0.1	4.1	−5.095	0.000
Fixed	Fatigue	6.0	4.8	7.4	−23.406	0.000
Fixed	Headache	0.9	0.2	3.7	− 6.546	0.000
Fixed	Hypoalbuminaemia	2.1	0.7	5.7	−7.141	0.000
Fixed	Hypokalaemia	7.1	4.7	10.7	−11.279	0.000
Fixed	Hyponatraemia	4.2	1.9	9.0	−7.481	0.000
Fixed	Increased alanine aminotransferase	1.3	0.2	8.5	−4.316	0.000
Fixed	Increased aspartate aminotransferase	2.7	1.5	4.7	−12.205	0.000
Fixed	Increased blood alkaline phosphatase	3.8	0.8	17.0	−3.843	0.000
Fixed	Increased blood bilirubin	2.3	1.0	5.5	−8.204	0.000
Fixed	Infusion-related reactions	0.7	0.1	3.6	−5.983	0.000
Fixed	Interstitial lung disease	0.8	0.4	1.9	−11.634	0.000
Fixed	Intestinal perforation	3.8	0.2	40.3	−2.232	0.026
Fixed	Malaise	1.0	0.4	2.6	−9.602	0.000
Fixed	Nasopharyngitis	0.6	0.1	4.1	−5.095	0.000
Fixed	Nausea	5.8	4.7	7.2	−23.716	0.000
Fixed	Neutropenia	5.1	1.9	12.9	−5.684	0.000
Fixed	Oedema	0.8	0.2	3.0	−6.832	0.000
Fixed	Pneumonitis	1.9	0.8	4.2	−9.203	0.000
Fixed	Pyrexia	1.5	0.7	3.5	−9.631	0.000
Fixed	Rush	0.4	0.0	6.5	−3.840	0.000
Fixed	Stomatitis	1.3	0.4	4.5	−6.745	0.000
Fixed	Upper respiratory tract infection	1.3	0.2	8.6	−4.303	0.000
Fixed	Vomiting	2.5	1.8	3.6	−19.390	0.000
Fixed	Weight decreased	2.1	0.8	5.4	−7.623	0.000
Random	Anemia	15.0	9.6	22.8	−6.654	0.000
Random	Decreased appetite	3.3	1.5	6.9	−8.426	0.000
Random	Decreased neutrophil count	20.3	14.5	27.6	−6.646	0.000
Random	Decreased platelet count	8.6	6.4	11.5	−14.412	0.000
Random	Decreased white blood cell count	10.3	7.2	14.5	−10.855	0.000

**Table 3:** The adverse event rates (all grades) and 95% confidence interval of fixed model and random model in single-arm trials

Model	Group by	All grade				
		Event rate (%)	Lower limit (%)	Upper limit (%)	Z-Value	p-Value
Fixed	Abdominal pain	13.6	10.7	17.3	−12.907	0.000
Fixed	Alanine aminotransferase increased	17.9	14.8	21.4	−13.234	0.000
Fixed	Cholangitis	5.5	1.8	15.9	−4.765	0.000
Fixed	Cough	16.5	13.5	20.1	−13.286	0.000
Fixed	Dizziness	13.3	8.2	20.9	−6.768	0.000
Fixed	Dysgeusia	15.1	11.0	20.5	−9.120	0.000
Fixed	Dyspnea	14.8	7.6	26.9	−4.566	0.000
Fixed	Electrocardiogram QT prolonged	6.7	3.9	11.5	−8.749	0.000
Fixed	Epistaxis	11.6	7.6	17.3	−8.546	0.000
Fixed	Hypoalbuminaemia	12.3	8.5	17.4	−9.382	0.000
Fixed	Hypokalaemia	15.3	11.9	19.4	−11.667	0.000
Fixed	Hyponatraemia	8.2	4.8	13.6	−8.334	0.000
Fixed	Increased alanine aminotransferase	9.0	4.3	17.6	−5.848	0.000
Fixed	Increased blood alkaline phosphatase	11.3	5.8	21.1	−5.473	0.000
Fixed	Increased blood bilirubin	4.2	2.4	7.4	−10.347	0.000
Fixed	Infusion-related reactions	1.7	0.6	4.7	−7.536	0.000
Fixed	Intestinal perforation	3.8	0.2	40.3	−2.232	0.026
Fixed	Malaise	24.3	20.6	28.5	−10.369	0.000
Fixed	Nasopharyngitis	10.3	6.6	15.8	−8.675	0.000
Fixed	Nausea	72.3	69.8	74.6	15.856	0.000
Fixed	Neutropenia	6.4	2.7	14.5	−5.800	0.000
Fixed	Oedema	12.2	8.8	16.7	−10.609	0.000
Fixed	Pneumonitis	8.2	5.6	11.9	−11.503	0.000
Fixed	Pyrexia	20.4	17.0	24.3	−11.903	0.000
Fixed	Rush	13.0	8.0	20.5	−6.852	0.000
Fixed	Stomatitis	22.9	17.9	28.7	−7.797	0.000
Fixed	Upper respiratory tract infection	11.1	6.7	17.9	−7.319	0.000
Fixed	Weight decreased	14.3	10.3	19.5	−9.352	0.000
Random	Abdominal distension	19.0	6.4	44.7	−2.296	0.022
Random	Alopecia	35.6	29.8	41.8	−4.422	0.000
Random	Anaemia	37.2	32.2	42.5	−4.648	0.000
Random	Constipation	26.9	22.4	31.8	−8.174	0.000
Random	Decreased appetite	44.3	35.0	54.1	−1.135	0.256
Random	Decreased lymphocyte count	12.6	7.3	20.8	−6.325	0.000
Random	Decreased neutrophil count	36.4	30.6	42.7	−4.131	0.000
Random	Decreased platelet count	29.0	25.0	33.4	−8.565	0.000
Random	Decreased white blood cell count	26.1	22.4	30.0	−10.345	0.000
Random	Diarrhoea	29.5	25.5	33.9	−8.382	0.000
Random	Ejection fraction decreased	3.0	0.6	14.1	−4.075	0.000
Random	Fatigue	38.5	32.4	45.0	−3.426	0.001
Random	Headache	14.6	8.4	24.2	−5.535	0.000
Random	Increased aspartate aminotransferase	19.5	14.8	25.2	−8.386	0.000
Random	Interstitial lung disease	7.5	4.5	12.1	−9.151	0.000
Random	Vomiting	39.2	33.8	44.9	−3.698	0.000

### Efficiency

The characteristics of overall survival condition of each study were summarized in Table 4. Median PFS is the time from the first treatment to the time disease progressing or dead without any recorded progress. The PFS of patients with breast cancer was 9.0-22.1 months, 3.0-8.3 to gastric cancer and 4.1-11.9 to other HER2-positive cancers.

**Table 4:** Summary of the efficiency information of all involved articles

Autor	Year	Pathological pattern	TTR (mo)	DOR (mo)	PFS (mo)	OS (mo)	follow-up (mo)	ORR (%)
**Breast cancer related studies**								
Modi	2019	HER2-positive meta- static breast cancer	1.6 (1.4 - 2.6)	14.8 (13.8 - 16.9)	16.4 (12.7 - NE)	–	11.1 (0.7 - 19.9)	60.9 (53.4 - 68.0)
Tamura	2019	HER2-positive breast cancer	1·6 (1·4 - 2·8)	20·7	22·1	–	9·9 (6·9 - 14·3)	59·5 (49·7 - 68·7)
Modi	2020	Advanced/ metastatic HER2-low–expressing breast cancer	2.6 (1.3 - 3.1)	10.4 (8.8 - NE)	11.1	29.4 (12.9 - 9.4)	–	37.0 (24.3 - 51.3)
Modi	2022	HER 2-low advanced breast cancer	2.73	10.7	9.9 (9.0-11.3)	23.4 (20.0-24.8)	18.4 (17.7-18.9)	52.3 (47.1 - 57.4)
Cortés	2022	HER2-positive metastatic breast cancer	–	14.3 (0.7-29.8)	NR (18.5 - NE)	–	16.2 (0 - 32.7)	79.9 (74.3-84.4)
**Digestive system cancer related studies**								
Shitara	2020	HER2-positive advanced gastric cancer	–	11.3 (5.6 - NE)	5.6 (4.3 to 6.9)	12.5	≥4 weeks after the initial response	51
Shitara	2019	HER2-positive gastric or gastro-oesophageal junction cancer	1·4 (1·3 - 1·6)	11.3	5·6 (3·0 - 8·3)	–	5·5 (2·8 - 13·1)	43·2 (28·3 - 59·0)
Siena	2021	HER2 IHC3+ or IHC2+ and ISH-positive metastatic colorectal cancer	–	–	6∙9 (4∙1 - NE)	5.4 (4.1-8.3)	4·1 (2.9 - 5.7)	45·3 (31·6 - 59·6)
		HER2 IHC2+ and ISH-negative metastatic colorectal cancer			–	–	1.4 (1.2 - 3.3)	
		HER2 IHC1 + metastatic colorectal cancer			–	–	2.0 (1.4 - 3.0)	
**Other studies**								
Doi	2017	Breast or gastric or gastro-oesophageal carcinomas	3	–	–	–	6·7	43 (23·2 - 65·5)
Li	2022	HER2-mutant non-small-cell lung cancer	1.5 (1.2 - 9.3)	9.3 (5.7 - 14.7)	8.2 (6.0 - 11.9)	17.8 (13.8 - 22.1)	13.1 (0.7 - 29.1)	55 (44 - 65)
Tsurutani	2020	HER2-expressing non-breast/non-gastric or HER2-mutant solid tumors	1.4 (1.4 - 2.9)	13.4	7.2 (4.8 - 11.1)	23.4 (15.6 - NE)	7.8 (0.1 - 28.6)	28.3 (17.5 - 41.4)

*Abbreviation: NE* not estimable, *NR* not reached, *OS* overall survival, *ORR* objective response rate, *PFS* progression-free survival, *TTR* time to response, *DOR* duration of response, *Mo* months

The DOR for breast cancer and other HER2-positive cancers were 0.7-29.8 and 5.6-14.7 months, respectively. The median ORR was 37-79.9% in patients with breast and gastric cancer and 28.3-55% in patients with other HER2-positive cancers. TTR is various in different study even with similar disease, the median ranged from 1.4 to 2.73 months among all patients. The median OS was reported in fewer studies, 23.4-29.4 months in patients with breast cancer and 5.4-23.4 months in patients with other non-breast/non-gastric solid cancer.

## Discussion

For curing patients with HER2-positive carcinoma, especially breast and gastric cancer, DS-8201a is a newly developed ADC, having combination of the HER2-targeted antibody and a topoisomerase I inhibitor, with great potency [23, 27, 33]. As patients with HER2-positive cancer still suffer from disease progression after using medicines according to guidelines, new drugs are in urgent demand [19]. This is the first study that explored the efficiency and safety of DS-8201a in treating HER2-positive cancer. The most common adverse event of DS-8201a is associated with gastrointestinal system and blood system. The ORR is higher and the time of PFS is longer in patients with breast and gastric cancer.

According to the pooled results, treated by DS-8201a resulted in an acceptable safety profile. The most common AEs mainly related to gastrointestinal and hematological system. In all grades, nausea, decreased appetite, vomiting, fatigue, anemia, decreased neutrophil count, alopecia and diarrhea had rates larger than 30%. In grade 3 or more, only decreased neutrophil count, anemia and decreased white blood cell count happened with relatively high rate. Compared with other anti-HER2 drugs like trastuzumab, pertuzumab and trastuzumab emtansine, which can lead to cardiac dysfunction and pulmonary toxicity, the AEs of DS-8201a are different and in high grades AEs are mainly related to hematological system [9, 21]. In addition, drug-related interstitial lung disease and pneumonia are life-threatening AEs despite their low incidence [22, 24, 25]. For patients suspected to have these AEs, treatment with DS-8201a should be interrupted pending further evaluations, like pulmonologist consultation, blood culture, high-resolution computerized tomography, et al. With early detection of symptoms, discontinuation, or reduction of DS-8201a use, and timely systemic corticosteroids, these life-threatening AEs may be effectively reduced [24, 28]. The relative safety of DS-8201a may due to its stabilization in plasma, as the cleavage of its linker needs lysosomal enzymes, which are sufficient in tumor cells and lack in plasma [19].

DS-8201a has high potency for HER2-positive cancers. The effect of DS-8201a for patients with HER2-positive breast and gastric carcinoma had been proved in included studies, in which a large proportion of patients had objective response to DS-8201a (ORR 37-79.9%). Compared with previous HER2-targeted agents, including margetuximab, neratinib, trastuzumab emtansine and lapatinib, the efficiency of DS-8201a was higher. For example, studies like SOPHIA, NALA, TH3RESA, EMILIA for breast cancer and GATSBY and TyTAN for gastric cancer used other HER2-targeted agents and gained ORR ranged from 16 to 32.8% [2, 15, 34–37]. For HER2-positive breast cancer, the recommended first-line neoadjuvant treatment is trastuzumab plus pertuzumab and a taxane, and the second-line therapy is ADC trastuzumab emtansine [38]. In comparison to previous study that used these neoadjuvant therapies for breast cancer, having ORR ranged from 40 to 60%, the ORR of DS-8201a was comparative [2, 39, 40]. In comparison with other recent therapies for HER2-positive breast cancer, the PFS of DS-8201a for breast cancer was longer (< 10 vs. 9.9-22.1 months) [22, 26, 27, 29]. These results indicated that DS-8201a had durable antitumor activity to HER2-positive cancer, especially breast cancer.

Though the ORR and PFS of patients with other kinds of solid tumor was relatively lower (17.5-65.5%, 4.1-11.9 months), conclusions could not be drawn due to the insufficient sample size. Larger studies are warranted to determine the potency of DS-8201a for HER2-amplified cancers. The variation of efficiency among different HER2-positive cancers may be due to different HER2 expression level in these cancers since many studies have proven the negative correlation between HER2 expression and cancer prognosis. The potency of DS-8201a to other HER2-mutated cancers may be mainly due to its high drug-to-antibody ratio and cytotoxic bystander effect [24].

In addition to higher ORR, PFS and OS, DS-8201a also offers more treatment options for patients who are resistant to previous anti-HER2 drugs. The resistance rate of using trastuzumab alone ranged from 66 to 88% and that of combination therapy was 20 to 50%. Even in patients with response, the one-year disease progression rate was high [41–43]. Many hypothesis reasons had been denounced, like the decrease, heterogeneous expression, or mutation of the out-membrane HER2, alternation of the proteinsides related to drug efflux and resistance to the intro-cellular drug payload [44, 45]. In included studies DS-8201a was still effective to patients previously treated by trastuzumab, pertuzumab or trastuzumab emtansine, and this may due to different pharmaceutical properties, including the potency of topoisomerase I inhibitor, the higher membrane permeability, bystander killing effect and larger drug-to-antibody ratio (7-8) of DS-8201a [17, 46].

There still are some limitations in this study and leaded to the high heterogeneity. Firstly, the dose of DS-8201a is 5.4 or 6.4 mg per kilogram of body weight, and for insufficient data subgroup analysis was not available. Secondly, patients included were heterogeneous with different kinds of HER2-positive tumors and differently prior treatments, which required more available research to address. Meanwhile, we included more than 50 kinds of symptoms reported in different research and it also contributed to the high heterogeneity. Lastly, no internal comparison was made to explore the efficiency of DS-8201a more directly. Thus, larger random control studies are required to assess the potency of DS-8201a.

In conclusion, DS-8201a plays an active role in treating HER2-positive cancers. The most common AEs of DS-8201a were related to gastrointestinal and hematological system. Decreased neutrophil count, anemia and decreased white blood cell count usually occur with high grades. More studies are required for exploring the ability of DS-8201a using alone or in combination with other drugs and finding methods to reduce AEs.

## Supplementary Information

**Additional file 1.**

**Additional file 2.**

## Data Availability

The datasets used and/or analyzed during the current study are available from the corresponding author on reasonable request.

## Abbreviations

ADC: Antibody-drug conjugate
AEs: Adverse events
CI: Confidence interval
DOR: Duration of response
HER2: Human epidermal growth factor receptor 2
NE: Not estimable
NR: Not reached
ORR: Objective response rate
OS: Overall survival
PFS: Progression free survival
TTR: Time to response
