# Supplementary material for: Safety and efficacy profile of Trastuzumab deruxtecan in solid cancer: pooled reanalysis based on clinical trials
Source: BMC Cancer. 2022 Aug 26;22:923. doi: 10.1186/s12885-022-10015-6 (PMC9414434; doi:10.1186/s12885-022-10015-6)
Supplement: Supplementary file 1 — Additional file 1. [file 12885_2022_10015_MOESM1_ESM.docx]

Supplementary table 1. Scores of Methodological items for non-randomized studies

| **Author** | **Year** | A clearly stated aim | Inclusion of consecutive patients | Prospective collection of data | Endpoints appropriate to the aim of the study | Unbiased assessment of the study endpoint | Follow-up period appropriate to the aim of the study | Loss to follow up less than 5% | Prospective calculation of the study size | Total score |
| --- | --- | --- | --- | --- | --- | --- | --- | --- | --- | --- |
| Doi | 2017 | 2 | 2 | 2 | 2 | 2 | 1 | 2 | 0 | 13 |
| Park | 2020 | 2 | 2 | 2 | 2 | 2 | 2 | 2 | 2 | 16 |
| Shitara | 2019 | 2 | 2 | 2 | 2 | 2 | 1 | 2 | 1 | 14 |
| Tamura | 2019 | 2 | 2 | 2 | 2 | 2 | 2 | 2 | 2 | 16 |
| Modi | 2020 | 2 | 2 | 2 | 2 | 2 | 0 | 2 | 1 | 13 |
| Shitara | 2020 | 2 | 2 | 2 | 2 | 2 | 1 | 2 | 2 | 15 |
| Tsurutani | 2020 | 2 | 2 | 2 | 2 | 2 | 2 | 2 | 1 | 15 |
| Siena | 2021 | 2 | 2 | 2 | 2 | 2 | 1 | 2 | 2 | 15 |
| Li | 2022 | 2 | 2 | 2 | 2 | 2 | 2 | 2 | 2 | 16 |
| Modi | 2022 | 2 | 2 | 2 | 2 | 2 | 2 | 2 | 2 | 16 |
| Cortés | 2022 | 2 | 2 | 2 | 2 | 2 | 2 | 2 | 2 | 16 |

1. A clearly stated aim: the question addressed should be precise and relevant in the light of available literature 2. Inclusion of consecutive patients: all patients potentially fit for inclusion (satisfying the criteria for inclusion) have been included in the study during the study period (no exclusion or details about the reasons for exclusion) 3. Prospective collection of data: data were collected according to a protocol established before the beginning of the study 4. Endpoints appropriate to the aim of the study: unambiguous explanation of the criteria used to evaluate the main outcome which should be in accordance with the question addressed by the study. Also, the endpoints should be assessed on an intention-to-treat basis. 5. Unbiased assessment of the study endpoint: blind evaluation of objective endpoints and double-blind evaluation of subjective endpoints. Otherwise the reasons for not blinding should be stated 6. Follow-up period appropriate to the aim of the study: the follow-up should be sufficiently long to allow the assessment of the main endpoint and possible adverse events 7. Loss to follow up less than 5%: all patients should be included in the follow up. Otherwise, the proportion lost to follow up should not exceed the proportion experiencing the major endpoint 8. Prospective calculation of the study size: information of the size of detectable difference of interest with a calculation of 95% confidence interval, according to the expected incidence of the outcome event, and information about the level for statistical significance and estimates of power when comparing the outcomes
